# A Matched Case-Control Study to Identify the Risk Factors for Gestational Diabetes Mellitus in a Tertiary Care Hospital in Chennai

**DOI:** 10.7759/cureus.103652

**Published:** 2026-02-15

**Authors:** Mitun Ganesan, Akash R, Pitchai Kumaran E, Seenivasan P, Sathish Viswanathan, Vashisht SR, Prasanna Raman R

**Affiliations:** 1 Community Medicine, Stanley Medical College, Chennai, IND; 2 Community Medicine, Government Thoothukudi Medical College, Thoothukudi, IND

**Keywords:** conditional logistic regression, gestational diabetes mellitus, matched case-control study, pre-pregnancy bmi, risk factors

## Abstract

Background

Gestational diabetes mellitus (GDM), defined as impaired glucose tolerance first recognized during pregnancy, can lead to serious maternal and fetal complications. Therefore, this study aimed to identify its risk factors in a tertiary care hospital in Chennai.

Methodology

A hospital-based matched case-control study with age matching was conducted in a tertiary care hospital in Chennai from August 2023 to September 2024. Using the WHO single-step oral 75 g glucose tolerance test (OGTT), 103 cases and 103 controls were individually matched to diagnose GDM. A pretested structured questionnaire and an examination of medical records were used in the data collection process. The McNemar test and conditional logistic regression were used for statistical analysis. The results were presented as matched (mOR) and adjusted matched odds ratios (AmOR) with 95% confidence intervals.

Results

The majority of the 206 pregnant women in the study were between the ages of 22 and 30 (103 cases and 103 age-matched controls). Cases were more likely to be obese (BMI ≥23 kg/m² Asian cut-off). Age-matched univariable analysis (McNemar test) revealed a significant association between pre-pregnancy BMI ≥23 kg/m² and GDM (mOR = 5.71; 95% CI: 2.56-12.76). The most reliable independent predictor, according to multivariable conditional logistic regression, was pre-pregnancy overweight/obesity (AmOR = 7.01; 95% CI: 2.96-16.64).

Conclusion

The findings of this study suggest that routine BMI screening, targeted preconception counseling, and lifestyle modifications to avoid overweight and obesity among women of reproductive age are crucial tactics to lessen the burden of GDM and the difficulties it causes for mothers and fetuses.

## Introduction

According to the World Health Organization (WHO) and the International Federation of Gynaecology and Obstetrics (FIGO), hyperglycemia in pregnancy (HIP) is divided into three categories: pre-gestational diabetes, gestational diabetes mellitus (GDM), and diabetes in pregnancy (DIP). Women who have type 1, type 2, or other types of diabetes prior to pregnancy are said to have pre-gestational diabetes. According to the International Classification of Diseases (ICD-11), GDM is defined as any level of glucose intolerance that is initially identified during pregnancy, does not match diagnostic criteria for diabetes outside pregnancy, and often goes away after delivery. DIP is the term for hyperglycemia initially identified during pregnancy that satisfies the WHO criteria for diabetes in adults who are not pregnant [1-3].

Hyperglycemia during pregnancy was expected to have affected 23.0 million (19.7%) live births worldwide in 2024. Of these, 9.9% had diabetes throughout pregnancy, 11% had pre-gestational diabetes, and 79.2% had gestational diabetes (GDM) [1,4]. With an overall prevalence of 27.8%, the South-East Asia (SEA) region has the highest age-standardized prevalence of hyperglycemia during pregnancy, at 31.7% [1]. According to a meta-analysis, the pooled prevalence of GDM in Asia was 11.5% (95% CI: 10.9-12.1), with significant regional variation and an estimated prevalence of roughly 8% in India [5]. In field research carried out in Tamil Nadu as part of the "Diabetes in Pregnancy - Awareness and Prevention" initiative, the prevalence of GDM among 4151, 3960, and 3945 screened women was 17.8% in urban areas, 13.8% in semi-urban regions, and 9.9% in rural areas [3,6].

Even when postpartum glucose levels seem normal, GDM represents an underlying metabolic imbalance that raises the long-term risk of type 2 diabetes and cardiovascular disease. Pregnant women who experience hyperglycemia are also at a higher risk of developing gestational diabetes mellitus in subsequent pregnancies. Pre-eclampsia, obstructed or protracted labor, cesarean birth, postpartum hemorrhage, infection, and retinopathy development are examples of maternal problems. Stillbirth, congenital defects, shoulder dystocia, birth trauma, neonatal hypoglycemia, and respiratory distress are among the fetal dangers [1,7,8]. 

Numerous unmatched case-control and cross-sectional investigations have revealed a variety of factors that contribute to the development of gestational diabetes mellitus (GDM) in pregnant women, including sociodemographic, genetic, obstetric, lifestyle, and comorbidity-related factors. Advanced maternal age, high pre-pregnancy BMI, a family history of diabetes mellitus, low maternal educational status, irregular physical activity prior to pregnancy confirmation, a history of GDM, a history of polycystic ovary syndrome (PCOS), a history of macrosomia birth, a history of cesarean section, and multiple pregnancies are all factors that contribute to GDM [9-15]. Given that the existing literature primarily relies on unmatched and cross-sectional study designs, and acknowledging the significant prevalence of GDM in urban settings along with its severe consequences for mothers and fetuses, this study was undertaken to identify independent predictors of GDM after controlling for age through matching and multivariable conditional logistic regression.

## Materials and methods

A matched case-control study with a hospital-based design was undertaken at the tertiary care hospital, Department of Obstetrics and Gynaecology, Chennai, Tamil Nadu, between August 2023 and September 2024. During this period, all pregnant women attending antenatal outpatient clinics or admitted to antenatal wards were subjected to routine screening for gestational diabetes mellitus (GDM). The diagnosis of GDM was made in accordance with nationally recognized guidelines that were in line with the WHO single-step protocol, which called for administering 75 g of oral glucose regardless of when the last meal had been consumed. Universal screening was advised for all pregnant women at their first prenatal visit, and repeat testing was recommended at 24-28 weeks of gestation if the initial result was negative. According to technical and operational guidelines for diagnosis and management of GDM by the National Health Mission, Maternal Health Division, Ministry of Health and Family Welfare, Government of India, a two-hour plasma glucose value greater than 140 mg/dL, measured using a plasma-calibrated glucometer, was considered diagnostic [3]. 

Women identified with GDM during the study period were enrolled as cases through consecutive recruitment until the predetermined sample size was attained. For each case, a corresponding control was selected from the same clinical setting and individually matched for age. Controls consisted of pregnant women with normal glucose values and were recruited from antenatal clinics or wards on the same day as the respective case. Equal representation of cases and controls was ensured by maintaining a 1:1 ratio throughout the study.

Participants with a prior diagnosis of diabetes mellitus (type 1 or type 2), disorders known to influence glucose metabolism, major visual, vestibular, neurological, sensory, or peripheral deficits, as well as those unable to participate in interviews due to intellectual disability or psychiatric illness, were excluded from both study groups.

Sample size calculation

The required sample size was estimated using the formula,

"$n = \frac{\left(Z_{1-\alpha/2} + Z_{1-\beta}\right)^2 \left[ P_1(1-P_1) + P_2(1-P_2) \right]} {(P_1 - P_2)^2}$"

Calculations were based on a confidence level of 95% (α = 0.05) and a statistical power of 80% (β = 0.20). The proportion of family history of diabetes among women with GDM (P1) was taken as 0.287, with Q1 = 0.713, while the corresponding proportion among women without GDM (P2) was assumed to be 0.129, with Q2 = 0.871. These proportions were derived from previously published findings by Kai Wei Lee et al among the Asian population [5]. Based on these assumptions, the final sample size was calculated as 103 participants per group (103 cases and 103 controls), yielding a total of 206 study participants.

Study tool and data collection procedure

After obtaining approval from the Institutional Ethical Committee, data were gathered through a pretested, validated, structured questionnaire using interviewer-administered face-to-face interviews after obtaining informed consent from the eligible study participants. The research instrument included three sections: (1) socio-demographic information of the participants including socioeconomic status assessed using Modified BG Prasad Scale - April 2024) [16]; (2) clinical information, such as comorbidities like polycystic ovarian syndrome (PCOS) and hypothyroidism, obstetric history, and family history of diabetes, which were extracted from medical records; and (3) maternal measurements, including pre-pregnancy weight (weight recorded within three months before conception was obtained during the first antenatal visit, which occurred within the first 12 weeks of gestation, based on the patient’s history as documented in the medical case records), height, and blood pressure. Medical records were only examined following de-identification. To ensure confidentiality, all personal identifiers, such as patient name, hospital registration number, address, phone number, and Aadhaar or other identity numbers, were eliminated before data extraction and analysis. The questionnaire was translated into Tamil and then back-translated into English to ensure its accuracy and consistency.

Data analysis

Microsoft Excel (Microsoft, Redmond, Washington) was used to enter the data, and SPSS version 16.0 (IBM Inc., Armonk, New York) was used for analysis. Frequencies and proportions were used to summarize categorical variables. The McNemar test was used in univariable matched analysis to assess relationships between individual exposures and gestational diabetes mellitus in light of the age-matched case-control design. The ratio of discordant pairs (b/c) was utilized to calculate the matched odds ratios (mORs) and 95% confidence intervals using simple conditional logistic regression. Only discordant matched pairs were included in the McNemar test and matched odds ratio computation.

The multivariable conditional logistic regression model included variables that were considered biologically plausible and those that demonstrated an association at p<0.20 in the univariable matched analysis (McNemar test). The regression analysis did not include the matching variable, age. Conditional logistic regression was used to estimate the final model, and the findings were displayed as adjusted matched odds ratios (AmORs) with 95% confidence intervals. A two-sided p-value <0.05 was used to determine statistical significance in the finally adjusted model.

## Results

The age distribution of the 103 cases and 103 controls was similar, indicating successful age matching, and the majority of individuals in both groups (132; 64.08%) were between the ages of 22 and 30. Most individuals had finished higher secondary or high school education, with high school education predominating among controls (34; 33.01%) and higher secondary schooling most frequently observed among cases (29; 28.16%). A significant portion of the participants - 89 (86.42%) of cases and 83 (80.59%) of controls - were homemakers. The upper-middle (80; 38.84%) and middle (55; 26.70%) socioeconomic strata comprised the majority of the study population, with similar distributions between cases and controls (Table 1).

**Table 1:** Socio-demographic characteristics of the study population among cases and controls (n=206)

Variables		Cases	Controls	Total
		Freq (%)	Freq (%)	Freq (%)
Age in years	19 to 21	11 (10.68)	11 (10.68)	22 (10.68)
	22 to 30	66 (64.08)	66 (64.08)	132 (64.08)
	>30	26 (25.24)	26(25.24)	52 (25.24)
Education	No formal schooling	7 (6.8)	4 (3.88)	11 (5.34)
	Primary	2 (1.94)	4 (3.88)	6 (2.91)
	Middle	15 (14.56)	9 (8.75)	24 (11.65)
	High	26 (25.24)	34 (33.01)	60 (29.13)
	Higher secondary	29 (28.16)	27 (26.21)	56 (27.18)
	Graduate/diploma	20 (19.42)	22 (21.36)	42 (20.39)
	Postgraduate	4 (3.88)	3 (2.91)	7 (3.4)
Occupation	Homemaker/unemployed	89 (86.42)	83 (80.59)	172 (83.5)
	Elementary occupation	4 (3.88)	3 (2.91)	7 (3.4)
	Craft and related trade workers	1 (0.97)	3 (2.91)	4 (1.94)
	Sales and service	1 (0.97)	3 (2.91)	4 (1.94)
	Clerical support workers	2 (1.94)	4 (3.88)	6 (2.91)
	Technicians and associate professionals	2 (1.94)	2 (1.94)	4 (1.94)
	Professionals	4 (3.88)	5 (4.86)	9 (4.37)
Modified BG Prasad socioeconomic status - April 2024	Upper	13 (12.62)	8 (7.77)	21 (10.19)
	Upper middle	40 (38.84)	40 (38.84)	80 (38.84)
	Middle	28 (27.18)	27 (26.21)	55 (26.7)
	Lower middle	22 (21.36)	23 (22.33)	45 (21.84)
	Lower	0 (0)	5 (4.85)	5 (2.43)
Total		103 (100)	10 3(100)	206 (100)

Type 2 diabetes mellitus (T2DM) was more common in cases (49; 47.57%) than in controls (38; 36.89%). Multigravida status was common in both groups with respect to obstetric history (cases: 60 (58.25%), controls: 57 (55.34%)). Pregnancy-induced hypertension (PIH) was more common in cases (26; 25.24%) than in controls (18; 17.48%). Compared to controls (6; 5.83%), a higher percentage of cases (12; 11.65%) reported a history of polycystic ovarian syndrome (PCOS). In both groups, maternal sleep disturbances were rare. Hypothyroidism was observed at similar rates among controls (11; 10.68%) and cases (9; 8.74%). Obesity was more common among cases (63; 61.17%) than among controls (42; 40.78%), according to Asian BMI criteria (Table 2).

**Table 2:** Clinical characteristics of the study population among cases and controls (n=206) T2DM - type 2 diabetes mellitus

Variables		Cases	Controls	Total
		Freq (%)	Freq (%)	Freq (%)
Family History of T2DM	Yes	49 (47.57)	38 (36.89)	87 (42.23)
	No	54 (52.43)	65 (63.11)	119 (57.77)
Obstetric history	Primigravida	43 (41.75)	46 (44.66)	89 (43.2)
	Multigravida	60 (58.25)	57 (55.34)	117 (56.8)
Pregnancy-induced hypertension	Yes	26 (25.24)	18 (17.48)	44 (21.36)
	No	77 (74.76)	85 (82.52)	162 (78.64)
PCOS	Yes	12 (11.65)	6 (5.83)	18 (8.74)
	No	91 (88.35)	97 (94.17)	188 (91.26)
Maternal sleep disturbance	Yes	7 (6.8)	8 (7.77)	15 (7.28)
	No	96 (93.2)	95 (92.23)	191 (92.72)
Hypothyroidism	Yes	9 (8.74)	11(10.68)	20(9.71)
	No	94 (91.26)	92 (89.32)	186 (90.29)
BMI (Asian classification)	Underweight	3 (2.91)	19 (18.45)	22 (10.68)
	Normal	15 (14.56)	32 (31.06)	47 (22.82)
	Overweight	22 (21.36)	10 (9.71)	32 (15.53)
	Obese	63 (61.17)	42 (40.78)	105 (50.97)
Total		103 (100)	103 (100)	206 (100)

Prior gestational diabetes mellitus (GDM) was notably more common in cases (21; 35.0%) than in controls (2; 3.51%) among multigravida subjects. The proportion of participants with a history of macrosomia was greater in cases (7; 11.67%) than in controls (1; 1.75%). Cesarean deliveries were performed in both groups, with a slightly higher proportion observed among cases (37; 61.67%) compared to controls (30; 52.63%). Cases had a higher frequency of prior low birth weight (LBW) deliveries (12; 20.0%) compared to controls (4; 7.02%) (Table 3).

**Table 3:** Characteristics of multigravida study participants among cases and controls (n=117) GDM - gestational diabetes mellitus

Variables		Cases	Controls	Total
		Freq (%)	Freq (%)	Freq (%)
GDM	Yes	21 (35)	2 (3.51)	23 (19.66)
	No	39 (65)	55 (96.49)	94 (80.34)
Macrosomia baby	Yes	7 (11.67)	1 (1.75)	8 (6.84)
	No	53 (88.33)	56 (98.25)	109 (93.16)
Cesarean section	Yes	37 (61.67)	30 (52.63)	67 (57.26)
	No	23 (38.33)	27 (47.37)	50 (42.74)
Low birth weight	Yes	12 (20)	4 (7.02)	16 (13.68)
	No	48 (80)	53 (92.98)	101 (86.32)
Total		60 (100)	57 (100)	117 (100)

Factors like schooling below the eighth grade, unemployment, family history of type 2 diabetes, multigravida status, previous PCOS, maternal sleep disturbance, and hypothyroidism did not exhibit significant relationships with gestational diabetes mellitus (GDM) in the age-matched univariable analysis using the McNemar test. On the other hand, GDM was strongly and substantially associated with a pre-pregnancy BMI ≥23 kg/m² (mOR = 5.71; 95% CI: 2.56-12.76; p<0.001) (Table 4).

**Table 4:** Age Matched-pair distribution and McNemar analysis of risk factors among matched cases and controls (206 samples, 103 age matched pairs) mOR - matched odds ratio; a - cases and control exposed, b - case exposed and control unexposed; c - case unexposed and control exposed; d - cases and control unexposed; T2DM - type 2 diabetes mellitus; PCOS - polycystic ovarian syndrome

Risk factor	Concordant pairs		Discordant pairs		mOR (95%CI)	McNemar p-value
	a	d	b	c		
Education - ≤ 8th standard	4	66	20	13	1.54 (0.76-3.10)	0.227
Occupation - Unemployed	74	5	15	9	1.67 (0.73-3.80)	0.226
Family history of T2DM	18	34	31	20	1.55(0.88-2.72)	0.127
Multigravida	37	23	23	20	1.15 (0.63-2.09)	0.648
PCOS	2	87	10	4	2.5 (0.78-7.98)	0.121
Maternal sleep disturbance	0	88	7	8	0.87 (0.32-2.42)	0.796
Hypothyroidism	3	86	6	8	0.75 (0.26-2.16)	0.594
Pre-pregnancy BMI ≥ 23	45	11	40	7	5.71 (2.56-12.76)	<0.001

The most important independent predictor of gestational diabetes mellitus (GDM) was found to be pre-pregnancy overweight or obesity (Asian BMI criterion ≥23 kg/m²). Even after controlling for family history of diabetes, parity, PCOS, maternal sleep disturbance, and hypothyroidism, women with a BMI ≥23 kg/m² prior to pregnancy had more than seven times the odds of developing GDM compared to those with a lower BMI (AmOR = 7.01; 95% CI: 2.96-16.64; p<0.001). On the other hand, adjusted matched odds ratios for variables like hypothyroidism, PCOS, multigravida status, maternal sleep disruption, and family history of type 2 diabetes mellitus were either lower or higher, although none of them reached statistical significance in the fully adjusted model. Notably, a history of PCOS was linked to a comparatively high adjusted matched odds ratio (AmOR = 3.86); however, this finding lacks statistical robustness due to the wide confidence range and borderline p-value (p=0.075) (Table 5).

**Table 5:** Independent predictors of gestational diabetes mellitus in an age-matched case–control study: multivariable conditional logistic regression analysis AmOR - adjusted matched odds ratio; T2DM - type 2 diabetes mellitus; PCOS - polycystic ovarian syndrome

Risk factor	AmOR	95%CI for AmOR		p-value
		Lower	Upper	
Family history of T2DM	1.6	0.80	3.21	0.184
Multigravida	1.43	0.68	2.99	0.343
PCOS	3.86	0.87	17.07	0.075
Maternal sleep disturbance	0.82	0.23	2.95	0.759
Hypothyroidism	0.36	0.10	1.29	0.116
Pre-pregnancy BMI ≥ 23	7.01	2.96	16.64	<0.001

## Discussion

With the global rise in gestational diabetes mellitus (GDM) and its detrimental effects on both maternal and neonatal health, pinpointing the most robust independent predictor of GDM is essential. Therefore, this study used a matched case-control design with paired analysis, in contrast to other research that mostly relied on unmatched case-control or cross-sectional methodologies.

A pre-pregnancy BMI of more than 23 kg/m² was the only significant independent predictor of GDM in the current study. This result is in line with previous research that indicates a higher pre-pregnancy or early-pregnancy BMI is a significant predictor of GDM, with overweight and obese women consistently showing a higher risk than those with normal BMI [5,9-11,13,17]. Even at BMI thresholds lower than those commonly employed in Western countries, evidence from Asian populations indicates that the risk of GDM rises [14,15]. National Family Health Survey (NFHS), demonstrating a strong dose-response association between rising BMI and GDM prevalence, corroborates the findings of Indian research, which also demonstrate significantly greater risk above Asian-specific BMI cut-offs [18,19]. After adjusting for potential confounders, some studies have not found an independent relationship between BMI and GDM [20,21]. This could be due to variations in study design, diagnostic standards, BMI classification, or underlying population characteristics.

The majority of previous research has consistently linked a positive family history of type 2 diabetes mellitus to an increased risk of gestational diabetes mellitus (GDM) [5,9-13,17,21,22]. Previous research indicates that women with a family history of diabetes may be at higher risk due to shared lifestyle variables, genetic susceptibility, and metabolic risk clustering. Therefore, variations in study design, population characteristics, sample size, or analysis methodology could account for the absence of a relationship between family history of T2DM and GDM in this study.

Polycystic ovarian syndrome (PCOS) was not found to be a predictor of gestational diabetes mellitus (GDM) in this study. This conclusion is at odds with a number of previous findings, such as those from China (Zhong et al.), Pakistan (Bibi et al.), and a meta-analysis (Lee et al.), all of which consistently showed that women with PCOS are more likely to develop GDM [5,14,23]. GDM susceptibility is expected to be increased by underlying metabolic and hormonal disorders in PCOS, but the lack of correlation in this study may be due to variations in demographic characteristics, sampling, or study design, highlighting the need for more research across diverse settings.

This study found no association between the number of pregnancies and gestational diabetes mellitus (GDM), which is in line with findings from Fatima et al., Chebrolu et al., Amiri et al., Feleke et al., Lee et al., and Mishra et al., who have all reported increased risk among women with many pregnancies, indicating that parity is a substantial risk factor [5,10,17,20,22,24,25]. Zhang et al., on the other hand, found that primigravida status was protective, which suggests that risk increases with consecutive pregnancies [13]. The mean age of the participants in this current study was only 27 years old, which may have lessened the cumulative metabolic impact of several pregnancies and, as a result, weakened any noticeable relationship between parity and the risk of GDM.

This study did not show any significant association between thyroid disorders and the occurrence of gestational diabetes mellitus (GDM), despite an analysis of NFHS data showing that women with thyroid disorders had a 1.29-fold higher risk of developing GDM compared to those without thyroid disorders [18]. While large national datasets can detect minor risks and may include more untreated or subclinical disease, the absence of a relationship in this study may be due to inadequate statistical power and dilution from mixed or treated thyroid disorders.

Strengths and limitations

Age matching successfully reduced maternal age confounding and improved internal validity, making this hospital-based age-matched case-control strategy ideal for identifying GDM risk variables. Reproducible case definitions and a decrease in misclassification bias were guaranteed by standardized diagnosis utilizing the WHO single-step 75 g glucose tolerance test (OGTT). The study's rigor was strengthened by the statistical justification of the sample size, which included explicit assumptions for effect size, confidence, and power. To improve measurement validity and reduce information bias, a pretested, validated, and culturally tailored questionnaire (translated into Tamil and back-translated) was used for data collection. The McNemar test and conditional logistic regression customized for matched data were used to establish analytical robustness. Lastly, informed consent processes and institutional ethical committee approval maintained ethical legitimacy.

The generalizability of this hospital-based study to community or primary/secondary care populations may be limited because it was carried out in a single tertiary care environment. Recall bias may have impacted self-reported obstetric, lifestyle, and medical history data, and selection bias may have occurred because controls were selected from the same hospital population. 

Reccomendations

Overall, the results show that the development of GDM in this population is mostly influenced by modifiable lifestyle-related factors, especially pre-pregnancy overweight and obesity. In order to lessen the burden of gestational diabetes mellitus and its related maternal and fetal problems, these findings highlight the necessity of preconception counseling, early risk assessment using routine BMI screening, and targeted lifestyle measures, including weight management prior to pregnancy.

## Conclusions

The independent risk factor for gestational diabetes mellitus (GDM) was found to be pre-pregnancy overweight and obesity (BMI ≥23 kg/m² Asian cut-off) in this age-matched case-control study conducted in a tertiary hospital. The mother's nutritional status before conception has been demonstrated to have a major impact on gestational glucose intolerance. Modifiable lifestyle factors, especially pre-pregnancy BMI, had a higher impact on GDM risk in this young, urban group than non-modifiable or medical factors, including parity, hypothyroidism, polycystic ovarian syndrome, or family history.

These findings emphasize the necessity of early risk stratification and preventative measures aimed at women of reproductive age, especially before pregnancy, from both public health and clinical viewpoints. The burden of GDM and its related maternal and fetal problems could be significantly decreased by routine BMI testing included in preconception and early antenatal care, together with counseling on weight management, a balanced diet, and physical activity.

## Appendices

QUESTIONNAIRE

**Title**: A matched case-control study to identify the risk factors for gestational diabetes mellitus in a tertiary care hospital in Chennai

**Participant ID:** ________ **Participant Type:** ☐ Case (GDM) ☐ Control (Non-GDM)

Section A: Socio-Demographic Details

1. What is your age in completed years? ______

2. What is your highest level of education level? ☐ No formal schooling  ☐ Primary   ☐ Middle   ☐ High school ☐ Higher secondary  ☐ Graduate / Diploma   ☐ Postgraduate

3. What is your current occupation? ☐ Homemaker / Unemployed   ☐ Elementary occupation  

☐ Plant and machine operators and assemblers    ☐ Craft and related trade worker

☐ Skilled agricultural, forestry and fishery workers  ☐ Sales / Service

☐ Clerical support worker   ☐ Technician / Associate professional ☐ Professional   ☐ Legislators, senior officials and managers

4. How many members are there in your family?

5. What is your total family income (monthly)?

6. What is your current marital status?

☐ Unmarried  ☐ Married  ☐ Widow/ widower

☐ Divorced  ☐ No response

Section B: Obstetric and Clinical History

7. Do you have a family history of diabetes mellitus?

☐ Yes ☐ No

If yes, relation: ☐ Parent ☐ Sibling ☐ Others

8. Have you been diagnosed with polycystic ovary syndrome? ☐ Yes ☐ No

9. Have you been diagnosed with Hypothyroidism?

☐ Yes ☐ No

10. Is this your first Pregnancy, if yes skip to question No.17 ☐ Yes ☐ No

11. Have you previously been diagnosed with GDM?

☐ Yes ☐ No

12. Have you been diagnosed with pregnancy-induced hypertension (PIH)? ☐ Yes ☐ No

13. Do you have Maternal sleep disturbance during pregnancy?

☐ Yes ☐ No

14. Have you ever delivered a macrosomia infant? ☐ Yes ☐ No

15. What was the previous mode of delivery? ☐ Normal Vaginal ☐ Caesarean section ☐ Assisted

16. Do you have a history of low-birth-weight infants in previous pregnancies? ☐ Yes ☐ No

Section C: Anthropometry

17. What was your weight before the current pregnancy (within 3 months of confirmation of pregnancy)? ______

18. Height (cm): ______

19. Pre-pregnancy BMI (Asian classification): ☐ Underweight   ☐ Normal ☐ Overweight ☐ Obese
